# Punish the Perpetrator or Compensate the Victim? Gain vs. Loss Context Modulate Third-Party Altruistic Behaviors

**DOI:** 10.3389/fpsyg.2017.02066

**Published:** 2017-11-28

**Authors:** Yingjie Liu, Lin Li, Li Zheng, Xiuyan Guo

**Affiliations:** ^1^School of Psychology and Cognitive Science, East China Normal University, Shanghai, China; ^2^National Demonstration Center for Experimental Psychology Education, East China Normal University, Shanghai, China

**Keywords:** third-party punishment, third-party compensation, loss context, empathic concern

## Abstract

Third-party punishment and third-party compensation are primary responses to observed norms violations. Previous studies mostly investigated these behaviors in gain rather than loss context, and few study made direct comparison between these two behaviors. We conducted three experiments to investigate third-party punishment and third-party compensation in the gain and loss context. Participants observed two persons playing Dictator Game to share an amount of gain or loss, and the proposer would propose unfair distribution sometimes. In Study 1A, participants should decide whether they wanted to punish proposer. In Study 1B, participants decided to compensate the recipient or to do nothing. This two experiments explored how gain and loss contexts might affect the willingness to altruistically punish a perpetrator, or to compensate a victim of unfairness. Results suggested that both third-party punishment and compensation were stronger in the loss context. Study 2 directly compare third-party punishment and third-party compensation in the both contexts, by allowing participants choosing between punishment, compensation and keeping. Participants chose compensation more often than punishment in the loss context, and chose more punishments in the gain context. Empathic concern partly explained between-context differences of altruistic compensation and punishment. Our findings provide insights on modulating effect of context on third-party altruistic decisions.

## Introduction

The existence of human society is based on social norms, and the ability to develop and enforce social norms is approximately one of the obvious features of the human species. However, the violation of social norms often occurred, e.g., violated fairness and justice. A notable fact is that norm compliance depends not only on the economic self-interest frequently served by collaboration and exchange conducted in a fairly manner, but also on the credible threat of consequences which are not welcome for defection (Spitzer et al., 2007). For instance, previous studies have shown that when people experienced an unfair economic distribution, they are willing to enforce the fair norms by punishing violator even at substantial personal costs (Boyd et al., 2003; Henrich et al., 2006). In lab researches, the ultimatum game (UG) has been frequently used to investigate such second-party punishment toward unfairness. In the UG, two players work together to split a sum of money. One player proposes how to split it and the other one responds (i.e., the proposer and the responder). The responder can either accept or reject the offer. The acceptance leads to the suggested division of money, whereas the rejection results in both players receiving nothing. Previous studies showed that responders were likely to reject unfair offers, especially for offers below 20% of the total (Güth et al., 1982; Camerer and Thaler, 1995), to make the unfair proposer get nothing and achieve the purpose of punishment.

It is worth noting that unfairness and injustice not only took place in the case of gains, but also in the case of losses. Given the fact that losses attracted more attention than equivalent gains (Peeters and Czapinski, 1990; Taylor, 1991) and the frame of gain or loss could influence people’s preferences and choices (Rothman and Salovey, 1997; Kühberger, 1998), some researchers examined whether second-party punishments toward unfair behaviors could be enhanced by the loss context, e.g., when proposer and responder needed to pay for a sum of money, and rejection to propose led to equal amount of monetary lose on both proposer and responder (Zhou and Wu, 2011; Guo et al., 2013). These works have revealed that subjective perception of social norm violations, fairness considerations as well as the second-party punishment (decision to reject) were modulated by the context of gain or loss. In particular, participants were likely to perceive higher unfairness level and have stronger desire to sanction social norm violations in the loss context than in the gain context (Zhou and Wu, 2011; Guo et al., 2013).

In real life, however, the maintenance of social norms was fulfilled not only through responses of the second-party, but also through actions by third parties. In fact, modern societies administer third-party behaviors to enforce social norms, by way of state-empowered legal system authorizing those unprejudiced third-party decision makers who are not influenced in a direct way by the norm violation and who do not have personal stake in the enforcement. For example, the distinctive third-party punishment was meted out in some social dilemmas when uninvolved third-parties witnessed injustices, which were frequently regarded as a transgression of normally held social norms such as the equal division rule (Messick, 1993, 1995). In order to study third-party behaviors toward norm violations, most researchers asked participants to observe some economic decision-making tasks being played between two persons. Among them, one popular task is the dictator game (DG) (Lotz et al., 2011; Leliveld et al., 2012; Hu et al., 2015), during which the dictator (proposer) was provided with an endowment and could make a decision about how to deal these out between him-/herself and the other person (recipient). Participants observed the DG as third-parties. In the third-party punishment tasks, participants might choose to punish the proposer following observation of an unfair distribution. When carrying out third-party punishments, third-parties voluntarily incur costs to punish violations of social norms. After inflicting punishment, violators to social norms were obliged to pay money, and the difference between the final outcome of the violators and their victims were decreased.

Notably, although studies on second-party punishment has proved that punishment toward unfairness could be modulated by the context of gain of loss, few research has considered how third-party actions toward unfairness might be affected by gain or loss context. When investigating third-party decision-making with economic games such as DG, most studies laid stress on economic game tasks in the gain domain (i.e., players distribute a sum of money as their gains) (Lotz et al., 2011; Leliveld et al., 2012; Hu et al., 2015), without taking into account the situations where individuals are obliged to share a certain amount of loss (i.e., players needed to pay for a sum of money). In the present study, we tried to explore whether third-party behaviors would be modulated in the loss context. We introduced the manipulation of gain and loss context to the study of third-party altruistic behavior, and it was expected that the loss context may increase third-parties’ sensitivity to norm violations, and improved their desires to carry out altruistic behavior.

There are some important discrepancies between second- and third-party responses toward social norm violations. As a second-party, the only choice to maintain fair norms is to reject (or punish) the proposer. But for a third-party decision maker, besides punishment there’s another way to deal with inequality through compensating victims of social norm violations (Schroeder et al., 2003; Prooijen, 2010).

However, third-party punishment and third-party compensation are likely to be motivated differently. The objective of punishment lies in just deserts, punishment can also be used to prohibit offenders from norm violations in the future (Carlsmith et al., 2002). As another measure coming to grips with inequality, compensation has a variety of underlying motivations. It is possible to recompense victims for the harm brought to them, and this behavior may be viewed as an endeavor to make the victim “whole” again (Schroeder et al., 2003). Thus, when investigating how gain or loss context might affect third-party maintenance of social norms, it is necessary to study both third-party punishment and third-party compensation simultaneously. Besides, another major discrepancy between third-party punishment and third-party compensation is that they aim to different targets. Third-party punishment is deployed to norm violators, while third-party compensation is targeting on victims of norm violations. Hence, the empathy for victims were peculiarly connected with the decision of third-party compensation. Empathy functions as the major proximal determinant of prosocial motivations (Lee and Murnighan, 2001), and it has been proved as being linked with third-party altruistic behaviors. In a study directly comparing punishment and compensation behavior (Leliveld et al., 2012), researchers found that high empathic people were inclined to provide compensation to victims, while low empathic people preferred to inflict punishment on perpetrators much often. Neuroimaging studies revealed subjects’ offers to low socio-economic status players were positively related with activations in left amygdala (neural resonance) and left fusiform cortex (trait empathy) (Carr et al., 2003; Pfeifer et al., 2008). A recent study also revealed that individual variations in empathic concern affects whether people are willing to compensate or to punish (Hu et al., 2015). As expected by the Empathy-Prospect model, empathy for victims of norm violations could be modulated by the context of gain and loss (Lee and Murnighan, 2001). Thus, it could be predicted that the perceptions of need together with the empathic responses and desires to offer help will become stronger for people who observe others’ losses rather than gains. Therefore, we anticipated that in the loss context, participants would compensate the recipient more often than they did in the gain context.

In the present research, we explored third-party observers’ desire to punish perpetrators or to compensate victims, and the impact of gain or loss context on third-party behaviors. In Study 1A, we firstly explored how context may affect third-party punishment, which was the most common third-party altruistic behavior. A modified third-party punishment game (Fehr and Fischbacher, 2004) was used in the study. Then, in order to investigate context effect on third-party compensation, a third-party compensation game (Leliveld et al., 2012) was played in both gain and loss contexts in Study 1B. Taken together, Study 1A and Study 1B explored how gain and loss contexts might affect the willingness to altruistically punish a perpetrator, or to compensate a victim of unfairness. Finally, the purpose of Study 2 was to replicate main findings of Study 1, and to test context effects on third-party altruistic behaviors when punishment and compensation were combined as two parallel options, so that participants were free to choose which type of altruistic behaviors they preferred in every trials. We took use of a third-party punishment/compensation game in both gain and loss contexts in Study 2, which made it possible for a direct comparison between context modulation on third-party punishment and third-party compensation.

In each of three experiments to be reported, three players took part in a game, among whom was the dictator (proposer, Player A), the recipient (B), and the observer (C). Participants always played as the observer, and they were made to believe that they would observe the history of two other players playing DG in gain context or loss context. Importantly, when observing an unfair distribution, in Study 1A, participants (observers) had a chance to punish proposer. While in Study 1B, participants were asked whether to compensate the recipient or not. And in Study 2, participants could decide whether they want to punish, to compensate, or to do nothing. We anticipated that gain and loss context regulates fairness preferences, and loss context should increase both third-party punishment and compensation. Moreover, we hypothesized that in the loss context, participants compensated the recipient more often than they did in the gain context. Previous studies concluded that empathic concern plays a major part in exerting some influence on people’s choice of either compensation or punishment (Leliveld et al., 2012; Hu et al., 2015). In our research, we measured empathic concern using one subscale of the Chinese version of Interactional Reactivity Index (IRI-C) (Zhang et al., 2010). We expected empathic concern remained to regulate the altruistic behavior (i.e., punishment or compensation) of people in the different contexts.

## Study 1A the Third-Party Punishment in Gain or Loss Context

### Method

#### Participants

Forty-eight students of East China Normal University [22 males, 26 females; mean age = 21.75 ± 2.75 (SD) years] participated in this experiment. This study was approved by the Research Ethics Committee of East China Normal University, all participants gave informed consent before performing the experiment and were paid according to outcomes from a random selection of 5% trials (i.e., two trials) plus a 15 RMB (approximately equal to 2.3 dollars) bonus.

#### Procedure

A modified third-party punishment game (Fehr and Fischbacher, 2004) was used in Study 1A. Before experiment, participants were told the rules of the game. They were told that they would observe histories of pairs of persons playing DG. Each pair of persons had jointly earned or lost 100 monetary units (MUs) by completing a task with same efforts in a previous study. Each MU equaled to 0.1 RMB (approximately 1.5 cents). Participants were then told that one of the two persons was randomly chosen as the proposer (Player A) and the other acted as the recipient (Player B). The proposer would make a proposal about how to allocate the jointly earned income (gain context) or how to share the loss (loss context). That is, in the gain context the two player would distribute the proceeds, and in the loss context the two player would bear the loss. The recipient would have no choices but to accept the proposal.

Then, similar to the classical third-party punishment game, participants were told that they would be endowed 50 MUs (Fehr and Fischbacher, 2004; Leliveld et al., 2012; Hu et al., 2015), and that they could decide to keep the MUs by themselves or to invest the MUs to punish Player A. If they chose to punish, for each MU they transferred, Player A’s payoff would decrease by three MUs. Participants would play with 48 pairs of persons, resulting in 48 rounds in total. In addition, they were informed that several rounds would be chosen randomly and that they would be paid according to their choices. An additional show-up fee of 15 RMB (approximately equal to 2.3 dollars) would also be paid.

All participants completed 48 trials, with 24 trials in either gain of loss context respectively. All trials were presented randomly. In each context, there were four fair trials (50:50) and 20 unfair trials (four trials for each distribution of 60:40, 70:30, 80:20, 90:10, and 100:0). This experimental design is similar to the design of previous studies (Leliveld et al., 2012; Hu et al., 2015). Generally, it was presumed that in the conditions of equal distribution, people would maintain this fair state and didn’t take third-party actions, so only unfair trials were focus of interest, and experiments usually consisted of more unfair trials than fair trials. This presumption held true in the current study (see results section for details). We presented each trial to the participants in a pseudo-random manner and the sequence of trials were counterbalanced between conditions. For unfair trials in the gain context, proposers proposed to take 60, 70, 80, 90, or 100 MUs; whereas in the loss context, they proposed to share 40, 30, 20, 10, and 0 MUs of lose. In the gain context, the distribution being proposed was presented by two horizontal red bars, whose lengths represented the ratio of distribution. For example, if the proposer proposed to take 60 MUs, then the upper bar (representing proposer) would be longer than the lower one (representing recipient), and their ratio in length was equal to 6:4. This visualization was similar to that used in the study of Hu et al. (2015). In the loss context, blue bars instead of red bars were used to represent share of loss between proposer and recipient, accordingly.

In each trial, participants were first presented with the proposal and were asked to choose between keeping the MUs (Keep) and punishing the proposer (Punish) by pressing keys (A or D) on keyboard. The two response keys were counterbalanced among the participants. As soon as participants responded, a red frame surrounding the selected choice would appear and lasted for 0.5 s. If participants chose to punish, they were further asked to choose a specific number of MUs they would invested among seven choices, which were 5, 10, 15, 20, 25, 30, or 35 MUs.

#### Trait Questionnaires

After the experiment, participants were asked to fill the Chinese version of IRI-C (Zhang et al., 2010), which includes 22 items and comprises four subscales: Perspective Taking, Fantasy, Empathic Concern, and Personal Distress. It is used to measure trait empathy. Participants need to rate on a five-point Likert scale ranging from 1 (almost never or never true) to 5 (almost always or always true).

### Results

#### Punishment Rate

**FIGURE 1:**
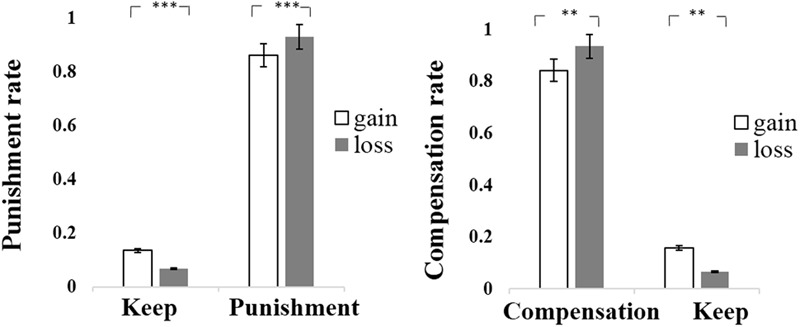
The decision rate of Punishment or Compensation of third-party chose between gain and loss context in study 1A or study 1B. ^∗∗^*p* < 0.01, ^∗∗∗^*p* < 0.001.

Statistical analyses were only focused on unfair trials. The reason we excluded fair trials was because that people never choose punishment in the fair trials (*M_punishment_* = 0). As a result, participants’ behaviors in fair trials had a variance of zero, and their behaviors in fair trials did not differ across contexts. Due to the fact that participants were always required to choose either Punish or Keep, the probability of choosing Keep is completely determined by the complementary probability of choosing “punish,” we used One-sample *t*-test to examine whether the punishment rate (probability of choosing “punish”) differed from chance (0.50). The results showed that the probability of punishment is significantly higher than 0.5 in the gain context [*M_Gain_* ±*SD* = 86.4 ± 10.8%, *t*(47) = 23.31, *p* < 0.001] and in the loss context [*M_loss_* ± *SD* = 93.1 ± 8.50%, *t*(47) = 34.97, *p* < 0.001]. Further, we used paired *t*-test to explore whether there was a difference in punishment rate between contexts. The results showed that punishment rate in the loss context was significantly higher than that in the gain context [*t*(47) = -4.65, *p* < 0.001, Cohen’s *d_z_* = -0.67 (see **Table 1** and **Figure 1**)], indicating that participants punished the proposer more often in the loss context compared with the gain context.

**Table 1:** Means, standard deviations of Decision rate and Transfer amount of participants choose Punishment, Compensation, or Keep and in gain and loss context.

	Choice of	Context	Decision rate	Transfer amount
	participants		(*M* ±*SD*)	(*M* ±*SD*)
Study 1A	Punishment	Gain	86.4 ± 11.1%	17.89 ± 5.04
		Loss	93.1 ± 8.3%	20.34 ± 4.14
Study 1B	Compensation	Gain	84.2 ± 22.3%	14.03 ± 5.34
		Loss	93.5 ± 8.8%	17.57 ± 4.78
Study 2	Punishment	Gain	72.7 ± 24.2%	18.02 ± 6.53
		Loss	23.6 ± 28.6%	12.65 ± 5.78
	Compensation	Gain	16.4 ± 20.8%	7.49 ± 4.18
		Loss	68.7 ± 30.3%	15.76 ± 6.92

#### Transfer Amount

**FIGURE 2:**
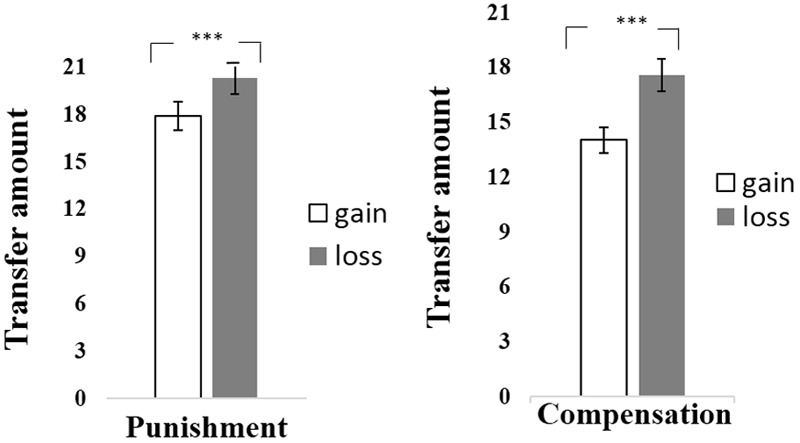
The mean of transfer amount of Punishment or Compensation between gain and loss context in study 1A or study 1B. ^∗∗∗^*p* < 0.001.

For each unfair trials, participants might select the amount of punishment if they decided to punish. And this amount of punishment could be set as 0 had participants decided not to punish. We calculated averaged transfer amount for each condition, and a paired *t*-test on transfer amount was conducted to explore whether there was difference in transfer amount between contexts. The results revealed that transfer amounts in the loss context was significantly higher than that in the gain context [*t*(47) = -4.47, *p* < 0.001, Cohen’s *d_z_* = -0.64; see **Table 1** and **Figure 2**], indicating that participants transferred more MUs in the loss context relative to the gain context.

Moreover, we also carried out a similar analysis on those unfair trials in which participants only decided to punish, in order to explore whether context still had an effect on transfer amount in case of participants having decided to punish. We excluded all unfair trials in which participants decided to keep, then calculated averaged transfer amount for each condition, and carried out a paired *t*-test. Results revealed the same context effect even when participants had already made a punishment decision. Transfer amounts in the loss context was significantly higher than that in the gain context [*M_Gain_* ±*SD* = 18.39 ± 5.58, *M_Loss_* ±*SD* = 20.37 ± 4.10; *t*(47) = -3.03, *p* < 0.01, Cohen’s *d_z_* = -0.40].

#### Regression Analysis

In order to examine how levels of unfairness and gain/loss context might affect third-party punishment. We conducted two regression analyses using contexts, unfairness level and their interaction to predict rate of punishment and transfer amount of punishment, respectively.

For punishment rate, regression analysis suggested that unfairness level, context and their interaction were included in the model. All had significant coefficients (for unfairness level, β = 0.013, *p* < 0.001; for context, β = 0.619, *p* < 0.001; and for their interaction, β = -0.007, *p* < 0.001). The results indicated that higher probability of punishment was related to higher level of unfairness, and the gain context. Importantly, the significant interaction between unfairness level and context suggested that third-party punishment toward unfairness was modulated by context of gain or loss. Specifically, in the gain context, level of unfairness predicted the rate of punishment (β = 0.013, *p* < 0.001), while in the loss context, level of unfairness had relative smaller effect (β = 0.006, *p* < 0.001).

For transfer amount of punishment, regression analysis indicated that unfairness level and context entered the model (for unfairness level, β = 0.567, *p* < 0.001; for context, β = 2.4, *p* < 0.05). The results indicated that higher transfer amount of punishment was related to higher level of unfairness, and the loss context. However, interaction between context and unfairness level was not significant.

#### Correlation between Behaviors and Empathy Concern

Correlation analyses were also conducted to test whether individual differences in empathic concern was related to altruistic behaviors. Correlations between participants’ empathic concern scores and their decision rate in each context were examined. Then, we correlated participants’ empathic concern scores with the difference of transfer amounts between the gain and the loss context. No significant result was revealed from either correlation analysis (*ps >* 0.165).

## Study 1B the Third-Party Compensation in Gain or Loss Context

### Method

#### Participants

Forty-six students of East China Normal University [20 males, 26 females; mean age = 21.04 ± 2.99 (SD) years] participated in this experiment. The participants were completely independent of the ones in Study 1A. This study was approved by the Research Ethics Committee of East China Normal University, all participants gave informed consent before performing the experiment and were paid according to outcomes from a random selection of 5% trials (i.e., two trials) plus a 15 RMB (approximately equal to 2.3 dollars) bonus.

#### Procedure

The procedure was almost the same as that in Study 1A, except that participants were asked whether he/she would compensate Player B or not. Participants still acted as third-party decision-makers (Player C) and could compensate the Player B with his/her endowment. If they chose to compensate the recipient, for each MU they transferred, Player B’s payoff would increase by three MUs.

#### Trait Questionnaires

After the experiment, participants were also asked to fill in the IRI-C scales.

### Results

#### Compensation Rate

Similar to the statistical method of Study 1A, we focused only on unfair trials, which was reasonable because of that people did not choose compensation in any fair trial (*M_compensation_* = 0). In Study 1B, the probability of choosing Keep was completely determined by the complementary probability of choosing compensate, so we used One-sample *t*-test to examine whether the compensation rate (the percentage of compensation decision compared in regard to all respective trials) was different from chance (0.50). The results showed that the probability of compensation is significantly higher than 0.5 in the gain context [*M_Gain_* ±*SD* = 84.2 ± 22.3%, *t*(45) = 10.41, *p* < 0.001] and in the loss context [*M_loss_* ±*SD* = 93.5 ± 9.1%, *t*(45) = 32.55, *p* < 0.001]. We used paired *t*-test to explore whether there was difference in compensation rate between contexts. The results showed that compensation rate in the loss context was significantly higher than that in the gain context [*t*(45) = *-*3.32, *p* < 0.01, Cohen’s *d_z_ = -*0.49; see **Table 1** and **Figure 1**], indicating that participants compensated the victims more often in the loss context compared with the gain context.

#### Transfer Amount

Similar to analyses in Study 1A, we firstly investigate transfer amounts with all unfair trials, setting compensation amount as 0 had participants decided not to compensate. A paired *t*-test revealed that the transfer amount in the loss context was significantly higher than that in the gain context [*t*(45) = *-*4.83, *p* < 0.001, Cohen’s *d_z_* = *-*0.71; see **Table 1** and **Figure 2**], indicating that participants transferred more MUs in the loss context relative to the gain context.

Secondly, we also carried out a similar analysis on those unfair trials in which participants only decided to compensate, in order to explore whether context still had an effect on transfer amount in case of participants having decided to compensate. Paired *t*-test revealed the same context effect even when participants had already made a compensation decision. Transfer amounts in the loss context was significantly higher than that in the gain context [*M_Gain_* ±*SD* = 14.66 ± 4.57, *M_Loss_* ±*SD* = 17.68 ± 4.93; *t*(43) = *-*4.79, *p* < 0.001, Cohen’s *d_z_* = *-*0.64].

#### Regression Analysis

In order to examine how levels of unfairness and gain/loss context might affect third-party compensation. We respectively conducted two regression analyses using contexts, unfairness level and their interaction to predict rate of compensation and transfer amount of compensation.

For compensation rate, regression analysis suggested that unfairness level, context and their interaction were included in the model. All had significant coefficients (for unfairness level, β = 0.010, *p* < 0.001; for context, β = 0.408, *p* < 0.01; and for their interaction, β = -0.004, *p* < 0.05). The results indicated that higher probability of compensation was related to higher level of unfairness, and the gain context. The significant interaction between unfairness level and context suggested that third-party compensation toward unfairness was modulated by context of gain or loss. Specifically, in the gain context, level of unfairness predicted the rate of compensation (β = 0.010, *p* < 0.001), while in the loss context, level of unfairness had relative smaller effect (β = 0.006, *p* < 0.001).

For transfer amount of compensation, regression analysis indicated that unfairness level and context entered the model (for unfairness level, β = 0.445, *p* < 0.001; for context, β = -0.9, *p* < 0.05). The results indicated that higher transfer amount of compensation was related to higher level of unfairness, and the loss context. However, interaction between context and fairness level was not significant.

#### Correlation between Behaviors and Empathy Concern

There was no significant results were detected between empathic concern and altruistic behaviors (*ps >* 0.157).

## Study 2 Punishment Versus Compensation in Gain or Loss Context

Study 1 showed that when people were able to punish norm violators (e.g., proposer) or compensate victims of injustice (e.g., recipient), more altruistic decisions would be made in the loss context compared to the gain context. However, in each experiment in Study 1, type of altruistic behavior that participants might choose was restricted, i.e., punishment or compensation. In order to further explore whether people had a preference for different types of altruistic behaviors in the gain or loss contexts, we conducted another experiment in which participants could decide which type of third-party altruistic behavior they want to carried out.

### Method

#### Participants

Seventy-five students of East China Normal University [34 males, 41 females; mean age = 21.27 ± 3.2 (SD) years] participated in this experiment. This study was approved by the Research Ethics Committee of East China Normal University, all participants gave informed consent before performing the experiment and were paid according to outcomes from a random selection of 5% trials (i.e., three trials) plus a 20 RMB (approximately equal to 3 dollars) bonus.

#### Procedure

The procedure was similar to those in Studies 1A and 1B, except that participants were asked to choose among three options: keeping the endowment to him/herself, or punishing Player A with his/her endowment, or Compensating Player B. If they chose to punish, for each MU they transferred, Player A’s payoff would decrease by three MUs; if they chose to compensate, for each MU they transferred, Player B’s payoff would increase by three MUs.

In addition, participants were told that they would play with 60 pairs of persons, resulting in 60 rounds in total. They were also informed that several rounds would be chosen randomly and that they would be paid according to their choices. An additional show-up fee of 20 RMB (approximately equal to 3 dollars) would also be paid.

All participants completed 60 trials, with 30 trials in either gain of loss context respectively. All trials were presented randomly. In each context, there were 4 fair trials (50:50) and 26 unfair trials (6 trials for distribution of 60:40, 70:30, 80:20; 4 trials for distribution of 90:10, 100:0). For unfair trials in the gain context, proposers proposed to take 60, 70, 80, 90, or 100 MUs; whereas in the loss context, they proposed to share 40, 30, 20, 10, and 0 MUs of lose.

In each trial, participants were first presented with the proposal and were asked to choose among keeping the MUs (Keep), punishing the proposer (Punish) and compensating the recipient (Help) by pressing keys (A, S, or D) on keyboard. The three response keys were counterbalanced among the participants. As soon as they responded, a red frame surrounding the selected choice would appear and lasted for 0.5 s. If participants chose to punish or compensate, they were further asked to choose the specific number of MUs they would transfer among seven choices, i.e., 5, 10, 15, 20, 25, 30, and 35 MUs.

#### Trait Questionnaires

After the experiment, participants were also asked to fill in the IRI-C scales.

### Results

#### Decision Rate

**FIGURE 3:**
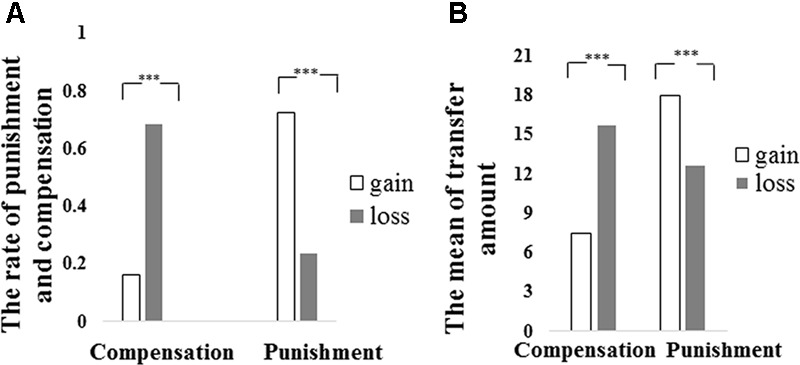
**(A)** The rate of Punishment and Compensation of third party chose between gain and loss context in Study 2. **(B)** The mean of transfer amount of Punishment and Compensation between gain and loss context in Study 2. ^∗∗∗^*p* < 0.001.

Similarly, the statistical analyses were only focused on unfair trials, because that participants did not choose punishment or compensation in any fair trial (*M_punishment_* = 0, *M_compensation_* = 0) and that neither punishment nor compensation in fair trials differed between gain and loss contexts. A repeated measure analyses of variances (ANOVAs) using a 2 (Contexts: Gain vs. Loss) × 2 (Choice: Punish vs. Compensate) design was conducted on participants’ decision rate (the percentage of punish/compensation/keep decision compared in regard to all respective trials). A significant main effect of Context was revealed [*F*(1,74) = 13.83, *p* < 0.001, $\eta_{p}^{2}$ = 0.16]. Specifically, participants chose to punish the proposer or compensate the recipient in the loss context more often than they did in the gain context (Gain: *M* ±*SD* = 44.6 ± 36.1%; Loss: *M* ±*SD* = 46.1 ± 36.9%). The main effect of Choice was not significant [*F*(1,74) = 1.78, *p* > 0.05]. Importantly, a significant interaction between Context and Choice was observed [*F*(1,74) = 149.27, *p* < 0.001, $\eta_{p}^{2}$ = 0.67]. *Post hoc* comparisons revealed that participants were more likely to punish the proposer than to compensate the recipient in the gain context (*p* < 0.001; see **Table 1** and **Figure 3A**), while they compensated the recipient more often in the loss context (*p* < 0.001; see **Table 1** and **Figure 3A**).

#### Transfer Amount

Similar to analyses in Study 1, we defined amount of punishment or compensation as 0 in those unfair trails if participants decided to keep. Averaged transfer amount for each condition was calculated. A 2 (Context: Gain vs. Loss) ^∗^ 2 (Choice: Punish vs. Compensate) repeated measures ANOVA on transfer amount revealed significant main effects of Context [*F*(1,74) = 4.25, *p* < 0.05, $\eta_{p}^{2}$ = 0.05] and Choice [*F*(1,74) = 9.86, *p* < 0.01, $\eta_{p}^{2}$ = 0.12]. Specifically, participants transferred more endowment in the loss context relative to the gain context (Gain: *M* ±*SD* = 12.76 ± 4.65; Loss: *M* ± *SD* = 14.21 ± 5.77; *p* < 0.05) and the endowment participants transferred to punish the proposer were more than those they transferred to compensate the recipient (Punish: *M* ±*SD* = 15.34 ± 7.47; Compensate: *M* ±*SD* = 11.63 ± 6.03, *p* < 0.05). A significant Context ^∗^ Choice interaction was also observed [*F*(1,74) = 43.44, *p* < 0.001, $\eta_{p}^{2}$ = 0.37]. *Post hoc* comparisons revealed that participants transferred more endowment for punishment in the gain context compared with the loss context, while they transferred more endowment for compensation in the loss context compared with the gain context. In the gain context amount of punishments exceeded that of compensations (*p* < 0.001; see **Table 1** and **Figure 3B**), and in the loss context larger amounts of compensation than punishment were observed (*p* = 0.06, see **Table 1** and **Figure 3B**).

Furthermore, we carried out a Generalized Estimating Equations (GEEs) on those unfair trials in which participants decided to punish or compensate, in order to explore whether context still had an effect on transfer amount in case of participants having decided to punish or compensate. Results revealed that the main effects of Context (Wald χ^2^ = 25.27, *p* < 0.001) and Choice (Wald χ^2^ = 14.54, *p* < 0.001) were significant. Participants transferred more in the loss context relative to the gain context (Gain: *M* ±*SD* = 16.50 ± 6.82; Loss: *M* ±*SD* = 18.53 ± 6.37) and the amount participants transferred to punish the proposer were larger than those they transferred to compensate the recipient (Punish: *M* ±*SD* = 19.01 ± 6.88; Compensate: *M* ± *SD* = 15.86 ± 6.03). Notably, the Context ^∗^ Choice interaction was also significant (Wald χ^2^ = 26.72, *p* < 0.001). Further analysis found that the difference of punishment amounts between contexts was not significant (Gain_punish_: *M* ±*SD* = 18.26 ± 6.23; Loss_punish_: *M* ±*SD* = 20.19 ± 7.71, *p* > 0.05), but the amount of compensation was larger in the loss context compared with the gain context (Gain_compensate_: *M* ±*SD* = 13.38 ± 6.76; Loss_compensate_: *M* ±*SD* = 17.38 ± 5.00, *p* < 0.001). In other words, participants transferred more endowment for punishment than compensation in the gain context (*p* < 0.001), and they transferred more endowment for compensation than punishment in the loss context (*p* < 0.05).

#### Regression Analysis

In order to examine how levels of unfairness and gain/loss context might affect third-party punishment and compensation. We conducted two regression analyses using contexts, unfairness level and their interaction to predict rate of punishment and compensation and transfer amount of punishment and compensation, respectively.

For punishment rate, unfairness level, context and their interaction were included in the model (for unfairness level, β = -0.010, *p* < 0.001; for context, β = 0.555, *p* < 0.001; and for their interaction, β = -0.014, *p* < 0.001). Higher probability of punishment was related to higher level of unfairness, and the gain context. Third-party punishment toward unfairness was modulated by the context. Specifically, level of unfairness had a relatively larger effect on rate of punishment in the gain context (β = 0.010, *p* < 0.001) compared to the loss context (β = -0.004, *p* < 0.01). For compensation rate, unfairness level, context and their interaction survived in the model (for unfairness level, β = 0.006, *p* = 0.06; for context, β = 0.17, *p* < 0.05; and for their interaction, β = -0.004, *p* < 0.05). Higher probability of compensation was related to higher level of unfairness, and the loss context. Third-party compensation toward unfairness was modulated by the context. Specifically, level of unfairness predicted the rate of compensation only in the loss context (β = 0.004, *p* < 0.05), but not in the gain context (β = -0.001, *p* > 0.05).

For transfer amount of punishment, unfairness level, context and their interaction were included in the model (for unfairness level, β = 0.460, *p* < 0.001; for context, β = 13.63, *p* < 0.001; and for their interaction, β = -0.282, *p* < 0.001). Larger transfer amount of punishment was related to higher level of unfairness, and the gain context. The effect of unfairness level on punishment amount was larger in the gain context (β = 0.460, *p* < 0.001) than in the loss context (β = 0.178, *p* < 0.001). For transfer amount of compensation, unfairness level, context and their interaction survived in the model (for unfairness level, β = 0.076, *p* < 0.05; for context, β = -9.67, *p* < 0.01; and for their interaction, β = 0.250, *p* < 0.001). Higher transfer amount of compensation was related to higher level of unfairness, and the loss context. The effect of unfairness level on compensation amount was larger in the loss context (β = 0.326, *p* < 0.001), than in the gain context (β = 0.076, *p* < 0.01).

#### Correlation between Behaviors and Empathy Concern

**FIGURE 4:**
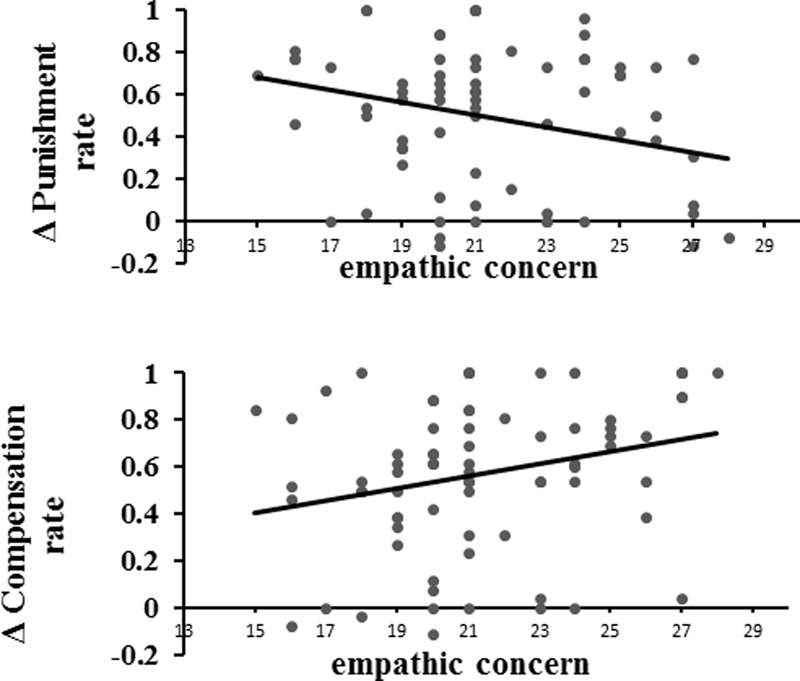
Correlation between empathic concern and Δ Punishment rate (the difference of decision rate for Punishment between the gain and loss context) and Δ Compensation rate (the difference of decision rate for Compensation between the loss and gain context).

A significant negative correlation was found between participants’ empathic concern and the difference of punishment rate across contexts (*r* = -0.262, *p* < 0.05; **Figure 4**), indicating that participants with low empathic tended to punish the proposer more often in the gain context compared with the loss context. Meanwhile, participants’ empathic concern was positively correlated with the difference of decision rate for compensation between contexts (*r* = 0.260, *p* < 0.05; **Figure 4**), indicating that participants with higher empathic concern compensated the recipients more often in the loss context compared with the gain context.

## Discussion

In the current research, we conducted three experiments to investigate third-party punishment and third-party compensation in gain and loss context. Studies 1A and 1B explored how gain and loss contexts might affect the willingness to altruistically punish a perpetrator, or to compensate a victim of unfairness, respectively. Consistent with our expectations, our results suggested that both third-party punishment and third-party compensation were stronger in loss compared to gain context. When observing the unfairness in loss context, people not only had higher rate of punishment or compensation, but also did they increase their level of punishment or compensation measured by transfer amounts. In order to directly compare third-party punishment with third-party compensation in both contexts, Study 2 instructed participants to choose one option from three instead of two alternatives. By doing so, we were able to investigate which type of third-party altruistic behavior might be preferred in the gain and loss context. To our knowledge, this is the first study investigating third-party punishment and compensation simultaneously. The current research might therefore contribute to the existing literature by introducing a paradigm that allows direct comparison between willingness of third-party punishment and willingness of third-party compensation. In line with results got from Studies 1A and 1B, Study 2 confirmed that people were more inclined to choose altruistic options (to punish or to compensate) in the loss context than the gain context. Moreover, Study 2 revealed that the likelihoods of both punishment and compensation were modulated by contexts. Participants tended to make more compensations than punishments in the loss context, and they also chose larger amount of transference for compensations than for punishments. In the gain context, on the contrary, participants tended to make more punishments than compensations, and they also transferred larger amount for punishments than for compensations. Besides, Study 2 revealed that third-party altruistic behaviors were related with individual’s empathic concern, indicating the later was correlated with individual’s between-context difference of decision rate for punishment or compensation. These results shed a new light on the literature of fairness and norms (Schroeder et al., 2003; Lotz et al., 2011) in which punishment and compensation have been studied in isolation, distinguishing and emphasizing the role of the two forms of reactions which were provided by human fairness system. The present research not only confirmed previous studies that people were willing to pay their own costs to enforce social norms (Fehr and Fischbacher, 2004; Lotz et al., 2011; Leliveld et al., 2012), but also provided the new evidences that according to the different fairness and norms contexts, human could reweigh social harms caused by unfairness and choose an altruistic behavior which is best suited to the current environment to achieve greater social value. Our results provided both theoretical and application value for the study of third-party altruistic behaviors.

Previous works have proved that third-party decision makers would choose to punish norm-violators even at their own expense (Fehr and Fischbacher, 2004), and some researches had indicated that people were willing to pay the same cost to compensate the victims (Lotz et al., 2011; Leliveld et al., 2012). Our research replicated these previous findings on third-party altruistic punishment and compensation, and suggested that third-party punishment and compensation could be stably observed even in a loss context. In Study 1A, rate of punishments in both contexts were significantly above chance. In fact, participants only chose to keep in few trials. Together with our findings from Study 1B, which revealed people preferred altruistic compensation to keep in both contexts, these results suggested that third-party altruistic behaviors (both punishment and compensation) were general reactions toward observed social norm-violations.

The detection of social norm violations, and subjective perception of magnitudes of such violations lead to decisions of third-party punishment or compensation. It has been proved that switching from the gain context to the loss context effectively changed subjective perception of social norm violations as well as fairness considerations (Zhou and Wu, 2011; Guo et al., 2013). Previous research also found participants were more likely to experience injustice and strong desire to sanction social norm transgressions in the loss context than in the gain context (Zhou and Wu, 2011; Guo et al., 2013). Other studies have found that negative stimuli such as loss, attracts more attention than equivalent positive stimuli such as gain (Peeters and Czapinski, 1990; Taylor, 1991). In our Study 1, the increased tendency to carry out third-party altruistic behavior in the loss context may result from people’s increased sensitivity toward social norm violations.

Despite that third-party punishment and compensation are both reactions to norm violations, these two behaviors seem to be driven by different motives. The objective of punishment lies in just deserts (Carlsmith et al., 2002), or to prohibit offenders from norm violations in the future (Carlsmith et al., 2002). In Study 2, participants had chance selecting either punishment or compensation as their preferred response to observed unfairness. We found that in the gain context, participants tended to make more punishments than compensations, and the intensity of punishments (measured by transfer amount) was also higher than that of compensations. Previous researches have demonstrated how the context of allocating gains may affect people’s focus of attention as well as their behavioral choices. For instance, people might be focused more on relationship issues in the gain context (Poppe and Valkenberg, 2003), probably because that differences in gains signaled more relational issues like respect and status (De Cremer, 2002). Moreover, when the people acted as a second-party in DG in the gain context, they were eager for apologies from the unfair proposer would overtake that for a financial compensation (De Cremer, 2010). Our Study 2 presented similar results by revealing people in the gain context were more inclined to punish perpetrators than in the loss context. We suggest this was due to that people have a desire to get perpetrators receive their “just deserts” or make them pay for it. Punishment might serve as a direct prove of people’s own power or status, and people believed that perpetrators deserved punishment proportional to the wrong committed. Because of this, people were more complied with social norms in the gain context, they wanted to give the perpetrator more just deserts and to maintain social norms from the perspective of long-term.

On the other hand, in Study 2, in the loss context participants tended to choose more third-party compensation and less third-party punishment than they did in the gain context. To recompense victims for the harm done to them, could also be viewed as an endeavor to make the victim “whole” again (Schroeder et al., 2003). In a previous research, De Cremer (2010) showed that if participants played as responders in a DG allocating losses, their subjective feeling would be more positive after getting financial compensations, rather than getting apologies from the proposer. That is to say, in the loss context, financial compensations might be considered as the most effective measures to heal the victim. In the current study, we also found that compared to the gain context, people tended to compensate victims in the loss context, which was in line with previous works. Moreover, the empathy-prospect model predicts that an observer’s reactions to severe losses would be particularly strong, so that the observation of someone facing a potential loss stimulated stronger perceptions of their need than someone facing a comparable gain, stronger perceptions of need stimulated stronger feelings of empathy, and stronger feelings of empathy stimulated stronger intentions to help (Lee and Murnighan, 2001). Taken together, our Study 1 suggested that people were more eager to carry out third-party altruistic behavior in the loss context. And our Study 2 specified that loss context stimulates two types of third-party altruistic behavior unequally. Specifically, people tended to prefer compensation than punishment in the loss context. We suggest the possible reason was that the loss context induced people considered the victims were more pitiful, and gave the victims more compensation in order to make them more “whole.”

Our research proved both third-party punishment and third-party compensation were modulated by context of gain or loss. In the Study 2, notably, both regression coefficients of unfairness level predicting punishment rate and punishment amount were larger in the gain context than in the loss context. On the contrary, both regression coefficients of unfairness level predicting compensation rate and compensation amount were larger in the loss context than in the gain context. Therefore, it indicated that gain/loss context modulated which type of third-party altruistic behavior was more sensitive to the degree of fairness norm violations. Higher level of unfairness was related much closer to increasing third-party punishment or increasing third-party compensation, in the gain context or loss context, respectively.

Both altruistic punishment and compensation were likely to be perceived as prosocial behavior. Researches showed that empathic concern induces the inclination to prosocial behavior (Davis, 1983; Eisenberg and Miller, 1987; Batson, 1991; Dovidio et al., 1991; Batson and Klein, 1995; Batson et al., 2003). The previous research found that empathic concern was related to third-party altruistic behaviors (Leliveld et al., 2012; Hu et al., 2015). In our Study 2, results showed that third-party altruistic behaviors were related with individual’s empathic concern. Specifically, empathic concern was correlated with individual’s between-context difference of decision rates for punishment or compensation. Research suggested that person’s possible gains or losses would influence the observer’s empathy and altruistic reactions, they would generate more intentions to help when observing losses (Lee and Murnighan, 2001). This was also similar to our behavior results. Other studies also found that people’s empathy toward those who had similar economic status induced them to provide assistance to their peers (Gino and Pierce, 2010). Neuroimaging studies revealed subjects’ offers to low socio-economic status players is positively correlated with the activation of left fusiform cortex, which is associated with trait empathy (Carr et al., 2003; Pfeifer et al., 2008). Together, researches confirmed that people may feel higher empathy for the inferior. We suggest this may be the reason why people would relatively improve their own compensation behavior when observing unfairness in the loss context.

According to previous research, following observation of injustice, people’s default reaction is inflicting punishment of the perpetrator (Gromet and Darley, 2009). However, considering the influence of context factors on the altruistic behaviors, especially in the loss context, compensation seemed to be the first-choice reaction toward observed norm violations. In future research, we might investigate how people respond to observed norm violations under time pressure in both gain and loss contexts. This would help to discuss whether people’s default response as a third-party observing norm violations might be different in the different contexts.

## Conclusion

The current research furnishes evidence that people have a tendency to punish a perpetrator or to compensate a victim as a third-party. The results confirmed that people were more inclined to choose altruistic options (to punish or to compensate) in the loss context than the gain context. The current research provided direct comparison between punishing and compensating behavior of third-party in the gain and loss contexts, suggesting that likelihoods of both punishment and compensation were modulated by contexts. Specifically, participants tended to make more compensations than punishments in the loss context, and also did they choose larger amount of transference for compensations than for punishments. Meanwhile, people tended to make more punishments than compensations in the gain context, and the amount of transference for punishments were larger than that for compensations. Our work revealed that people’s altruistic behaviors are influenced by the contextual factors, and that the difference of altruistic behavior choices between contexts might related with individual’s empathic concern. Our findings might be also helpful to a better understanding of peoples’ behaviors in the legal system or charity systems. For example, in real social life, people might sometime explain their eagerness in punishing criminals who made harm to others, or might they sometime express the same willingness in helping victims of crimes. Our study might help to understand why people decide to punish or compensate in different settings.

Besides, it was also appropriate to note some limitations of the current studies. Firstly, the current research made a direct comparison between third-party punishment and compensation, but our results only suggested how relative preference for punishment and compensation change as a function of context. This is because in the current study, participants always faced with exclusive options. So, it could be possible that participants might have a higher motivation to punish the proposers in the loss condition than in the gain condition, but they thought that it was even more important to compensate the victims in the loss condition. In the further studies, we may modify the task by allowing participant choosing more than one options at the same time, so that absolute instead of relative eagerness to punish or compensate could be investigated. It will provide more direct insights into the nature of third-party altruistic acts, and also enable us to evaluate recently developed theories of social preferences.

## Ethics Statement

This study was carried out in accordance with the recommendations of the Ethical Committee of East China Normal University with written informed consent from all subjects. All subjects gave written informed consent in accordance with the Declaration of Helsinki. The protocol was approved by the Ethical Committee of East China Normal University.

## Author Contributions

YL conceived the paper, ran statistical analyses and contributed to the manuscript. LL contributed to the manuscript. LZ conceived the paper and contributed to the manuscript. XG conceived the paper and contributed to the manuscript.

## Conflict of Interest Statement

The authors declare that the research was conducted in the absence of any commercial or financial relationships that could be construed as a potential conflict of interest.
